# Pharmacists and the Cocaine Snuff Habit

**Published:** 1914-06-13

**Authors:** 


### Pharmacists and the Cocaine Snuff Habit.

IN a letter appearing in a recent issue of the *Daily Mail* "An Anxious Relative" makes "an earnest appeal to the chemists of Great Britain and Ireland to refuse to supply youths with menthol snuff containing cocaine." The writer of the letter understands that the cocaine habit is growing among young men of the present day, and states that he has a young relative who is being ruined mentally, physically, and morally by the use of this drug; hence his appeal. Of the case the writer has in mind nothing can be said, but the experience of pharmacists is that the demand for cocaine is decreasing rather than increasing, and this seems to be corroborated by the state of the wholesale market for cocaine, which has been in a more or less depressed condition for a long time past—we might almost write, for years—as a result, it is commonly asserted, of the diminishing use of the drug. On the other hand, there is no lack of evidence that in the United States and France the cocaine habit has grown to alarming proportions. In this country, however, there is good reason to believe that pharmacists generally endeavour to discourage the use of preparations containing cocaine, unless, of course, they are ordered on the prescription of a medical practitioner.

